# Dynamic Modulation of Beta Band Cortico-Muscular Coupling Induced by Audio–Visual Rhythms

**DOI:** 10.1093/texcom/tgaa043

**Published:** 2020-08-05

**Authors:** Manuel Varlet, Sylvie Nozaradan, Laurel Trainor, Peter E Keller

**Affiliations:** The MARCS Institute for Brain, Behaviour and Development, Western Sydney University, Penrith, Australia; School of Psychology, Western Sydney University, Penrith, Australia; The MARCS Institute for Brain, Behaviour and Development, Western Sydney University, Penrith, Australia; Institute of Neuroscience (IONS), Université catholique de Louvain (UCL), Brussels, Belgium; Department of Psychology, Neuroscience & Behaviour, McMaster University, Hamilton, Ontario, Canada; McMaster Institute for Music and the Mind, McMaster University, Hamilton, Ontario, Canada; Rotman Research Institute, Baycrest Hospital, Toronto, Ontario, Canada; The MARCS Institute for Brain, Behaviour and Development, Western Sydney University, Penrith, Australia

**Keywords:** beta oscillations, cortico-muscular coupling, entrainment, rhythms, sensorimotor synchronization

## Abstract

Human movements often spontaneously fall into synchrony with auditory and visual environmental rhythms. Related behavioral studies have shown that motor responses are automatically and unintentionally coupled with external rhythmic stimuli. However, the neurophysiological processes underlying such motor entrainment remain largely unknown. Here, we investigated with electroencephalography (EEG) and electromyography (EMG) the modulation of neural and muscular activity induced by periodic audio and/or visual sequences. The sequences were presented at either 1 or 2 Hz, while participants maintained constant finger pressure on a force sensor. The results revealed that although there was no change of amplitude in participants’ EMG in response to the sequences, the synchronization between EMG and EEG recorded over motor areas in the beta (12–40 Hz) frequency band was dynamically modulated, with maximal coherence occurring about 100 ms before each stimulus. These modulations in beta EEG–EMG motor coherence were found for the 2-Hz audio–visual sequences, confirming at a neurophysiological level the enhancement of motor entrainment with multimodal rhythms that fall within preferred perceptual and movement frequency ranges. Our findings identify beta band cortico-muscular coupling as a potential underlying mechanism of motor entrainment, further elucidating the nature of the link between sensory and motor systems in humans.

## Introduction

Human movements spontaneously entrain to auditory and visual environmental rhythms, aligning in time to the rhythms in the absence of an individual’s intention to do so (Néda et al. 2000; Tognoli et al. 2007; Burger et al. 2013). Entrainment occurs with musical rhythms and others’ movements, for instance, and is critical for successful adaptation to the continuously changing constraints of everyday environments (Kelso 1995; Phillips-Silver et al. 2010). Here, we investigate the neurophysiological processes underlying the occurrence and strength of such motor entrainment to auditory and visual rhythms.

Neuroimaging studies have revealed that passive listening to, or observation of, auditory and/or visual rhythms activates motor areas in addition to sensory areas in the brain (Iacoboni et al. 1999; Grahn and Brett 2007; Chen et al. 2008), even if no overt movement is produced. Furthermore, dynamic amplitude modulations of 20-Hz beta band neural oscillations that underpin sensorimotor mechanisms align with the rhythms (Press et al. 2011; Fujioka et al. 2012, 2015). Similarly, the amplitude of beta band neural oscillations decreases during movement execution and rebounds when movement ends (Pfurtscheller 1981; Neuper and Pfurtscheller 2001). Despite growing evidence of the involvement of motor areas during passive listening and observation of rhythms, including brain network activity mediated through beta band oscillations, the neurophysiological processes underlying the production of motor responses aligned with external rhythms remain unclear.

This study combined electroencephalography (EEG) and electromyography (EMG) to better understand the contribution of beta band oscillations in motor entrainment to auditory and/or visual rhythms. These techniques were used together to examine the synchronization between beta band oscillations at cortical and muscular levels and test whether the degree of synchrony is dynamically modulated during passive rhythm listening and/or observation. Previous research has revealed that cross-spectral coherence between primary motor areas (M1) and muscular activity in the beta band during isometric contraction, which is taken to be a marker of motor control (Gross et al. 2000; Kilner et al. 2000; Bourguignon et al. 2017), is modulated by the presentation of unexpected visual and audio stimuli (Caetano et al. 2007; Hari et al. 2014; Piitulainen et al. 2015). Increased cortico-muscular coherence occurs a few hundred milliseconds after such stimulus presentation, suggesting an automatic activation of the motor system in response to environmental changes (Hari et al. 2014; Piitulainen et al. 2015).

Here, we investigated whether cortico-muscular beta coherence also changes dynamically in response to stimuli that repeat periodically and are therefore predictable, with coherence increasing prior to stimulus onsets as a possible mechanism of spontaneous motor entrainment. We examined the effects of unimodal visual and audio rhythmic stimuli as well as bimodal audio–visual rhythmic stimuli on EEG–EMG beta coherence when participants produced steady index finger flexion. Advantages of audio rhythms over visual rhythms, especially when discrete, have been shown in previous sensorimotor synchronization studies. This auditory advantage has been argued to originate from superior temporal processing for this modality (Repp 2003; Hove et al. 2012; Varlet, Marin, Issartel, et al. 2012). Advantages of bimodal audio–visual rhythms over unimodal rhythms have also been reported in behavioral studies as integration of information across sensory modalities can optimize event timing (Elliott et al. 2010, 2011). This suggests that if dynamic modulations of cortico-muscular coherence occur, they might be of greater magnitude for audio than visual rhythms and for bimodal than unimodal rhythms.

Visual and auditory stimuli were presented in either 1- or 2-Hz sequences, as the tempo of the rhythms strongly influences motor entrainment (Richardson et al. 2007; Varlet et al. 2020). Entrainment is superior for rhythms presented close to an individual’s preferred movement tempo, typically in the 2-Hz range, which may be related to optimal tempo for locomotion (MacDougall and Moore 2005; Large 2008; Todd and Lee 2015). 2 Hz is not only the preferred tempo for rhythm production but also for rhythm perception (Bauer et al. 2015), suggesting that if dynamic modulations of EEG–EMG beta coherence occur, their amplitude would be greater for 2-Hz than 1-Hz sequences.

## Materials and Methods

### Participants

Seventeen participants volunteered to take part in the study (15 females and 2 males, *M* = 26.75, SD = 7.66). All participants were right handed, had normal hearing, normal or corrected-to-normal vision, and provided written informed consent prior to the experiment, which was approved by the Human Research Ethics Committee at Western Sydney University.

### Apparatus

A wide bar load cell (HTC-Sensor TAL201) connected to an Arduino Duemilanove board (Arduino) via an amplifier shield (Load Cell/Wheatstone Amplifier Shield, RobotShop) was used to record the force exerted by right index finger of each participant. The Arduino board was connected to a MacBook Pro laptop (Apple) via USB. The load cell was calibrated for linearity and positioned on a custom support on the right arm of a chair on which the participant was seated. The chair was positioned in front of a 22-inch BenQ computer monitor that was used to display the visual stimuli with a refresh rate of 60 Hz. Audio stimuli were presented via insert earphones (ER-1, Etymotic Research).

### Stimuli

**Figure 1:**
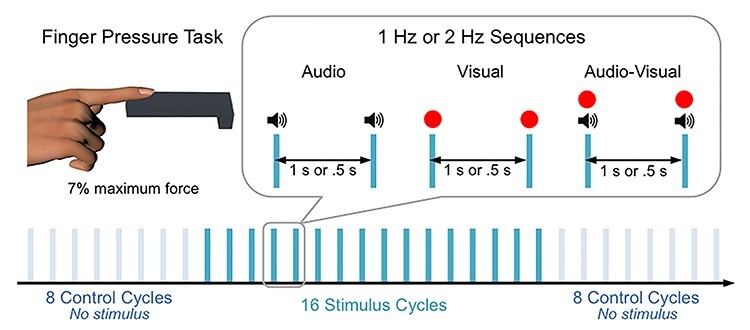
Illustration of the index finger pressure task and audio and/or visual sequences used in the current study.

Sequences of visual and/or audio stimuli at either 1 or 2 Hz were presented to participants during the experimental trials. Visual sequences consisted of red dots of 7 cm diameter (≈5° visual angle) presented on a black background at the center of the monitor for 5 frames (i.e., about 83 ms) every 1 s for 1-Hz trials and every 0.5 s for 2-Hz trials (see Fig. 1). Audio sequences consisted of 500-Hz sine tones presented for 5 frames (i.e., 83 ms, including 5-ms linear fade in and fade out) every 1 s for 1-Hz trials and every 0.5 s for 2-Hz trials at a comfortable listening level that was kept constant across participants (80 dB). All experimental trials started with 8 control cycles without a stimulus, followed by 16 cycles with audio and/or visual stimuli, and ended with 8 control cycles without a stimulus, as shown in Figure 1. The duration of the cycles was 1 s for 1-Hz trials and 0.5 s for 2-Hz trials. The onset of the audio and visual stimuli was at the middle of the stimulus cycles (i.e., 0.5 and 0.25 s for 1- and 2-Hz conditions, respectively). The experimental trials lasted in total 32 s for the 1-Hz condition and 16 s for the 2-Hz condition.

A letter detection visual task requiring constant vigilance was presented to participants on the monitor during experimental trials to make sure that they remained focused when the stimuli were presented (Schmidt et al. 2007; Varlet et al. 2017). A fixation cross was displayed at the center of the monitor throughout each trial, which alternated with letters, occurring briefly for 5 frames (i.e., about 83 ms) at random time intervals between 6 and 12 s (between 1 and 3 letters occurred in each trial). The participant was asked to remember the last letter that flashed on the monitor and say it aloud at the end of the trial under the monitoring of the experimenter.

### Procedure

On arrival, an information sheet was given to the participant before obtaining written consent. The sheet described the task as a letter detection task with visual, auditory and motor perturbations, requiring the participant to maintain a constant finger pressure while remembering letters that flashed at random times at the center of the monitor. This cover story was used to ensure that any modulations to the rhythmic sequences found in EEG and EMG data were unintended.

Once seated in the chair in front of the monitor, the participant was instructed to keep a constant pressure of the right index finger on the force sensor while keeping her or his forearm as still as possible (see Fig. 1). The participant was given a practice period before recording three 3-s pre-experimental trials in which she or he was instructed to produce the maximum possible pressure. The average of the maximum force in these 3 pre-experimental trials was taken as reference for the following experimental trials in which visual and/or audio sequences were presented.

Before each experimental trial, visual feedback based on the participant’s exerted finger force was displayed at the center of the monitor to allow the participant to (re)adjust the pressure to 7% of her or his maximum force. The value of 7% was selected in accordance with previous studies to ensure that the instructed force was in a range suitable for detecting cortico-muscular coherence while being maintained with minimal fatigue (Witte et al. 2007; Hari et al. 2014; Bourguignon et al. 2017). The visual feedback consisted of a vertical bar that varied in length in real time depending on the percentage of the instructed force produced by the participant. The bar was red and turned green when the exerted force equaled the instructed force ±5%, allowing the experimenter to start the trial. The participant was instructed to maintain the instructed pressure throughout the trial while keeping the eyes fixed on the fixation cross at the center of the monitor in order to perform the letter detection task (Hari et al. 2014; Piitulainen et al. 2015).

Each participant performed 72 experimental trials in total, with 12 trials for each of the 6 conditions tested—Modality (Audio, Visual and Audio–Visual) × Tempo (1 and 2 Hz). Each participant performed in total 12 blocks of 6 randomly ordered trials, one for each of the 6 experimental conditions. The participant was asked to keep the pressure on the force sensor constant for the entire block with the help of the visual feedback displayed between trials. The participant was asked to relax and rest between blocks. The total duration of the experiment was approximately 90 min, including EEG and EMG preparation.

### E‌EG and EMG Recording

EEG and EMG signals were recorded at a sampling rate of 2048 Hz using a Biosemi Active-Two system (Biosemi), which incorporates hardware low-pass filtering at one fifth of the sampling rate. EEG was recorded with 64 Ag–AgCl electrodes placed over the scalp of the participant according to the international 10/20 system. All electrodes were referenced to the Common Mode Sense (CMS) and their magnitude was kept below 50 μV. EMG of the flexor digitorum superficialis (FDS) involved in index finger flexion was recorded using a bipolar montage with 2 flat electrodes placed over the participant’s forearm (Hari et al. 2014; Varlet et al. 2017). Four additional flat electrodes placed above and below the right eye and the external corner of the left and right eyes were used to record ocular movements and eye blinks.

### E‌EG and EMG Analyses

#### Preprocessing

EEG data were first bandpass filtered using a fourth-order Butterworth filter with 0.1- and 100-Hz cut-off frequencies, notch filtered to remove 50-Hz power contamination, and then downsampled to 1000 Hz and segmented into 32- and 16-s trials. Channels containing excessive artifacts or noise were then interpolated with the neighboring channels (i.e., an average of one interpolated electrode per participant and never more than 3). An independent components analysis (FastICA), as implemented in Fieldtrip (Oostenveld et al. 2011), was used to remove blink artifacts and lateralized eye movements. Based on the visual inspection of the topography and time course, components corresponding to the blinks and lateralized eye movement were removed per participant. EEG data were then re-referenced to the average of all scalp electrodes.

EMG data were bandpass filtered using a fourth-order Butterworth filter with 10- and 195-Hz cut-off frequencies to remove motion artifacts and noise in line with previous EMG and EEG–EMG coherence studies (Piitulainen et al. 2015; Bourguignon et al. 2017), notch filtered to remove 50-Hz (and corresponding harmonics) power contamination, full-wave rectified (i.e., computation of absolute EMG values), and then down sampled to 1000 Hz and segmented into 32- and 16-s trials. Full-wave rectification is often used to examine EMG amplitude and cortico-muscular coupling, as it has been suggested to improve the detection of synchronization between EEG (or MEG) and EMG signals, although it should be noted that its actual benefit is still widely debated (Yao et al. 2007; Boonstra and Breakspear 2012; McClelland et al. 2012; Ward et al. 2013; Hari et al. 2014; Piitulainen et al. 2015).

These 2 EEG and EMG preprocessed datasets were then used to investigate 1) global amplitude modulations in EEG and EMG signals, as detailed below in broadband responses, and 2) specific modulations in the beta frequency band for the synchronization between EEG and EMG signals (EEG–EMG coherence), as well as their amplitude using time-frequency analyses, as detailed below.

#### Broadband Responses

For broadband responses, EEG signals were further filtered using a 4th order Butterworth bandpass filter with 0.3- and 30-Hz cut-off frequencies for visualization in the time domain of EEG evoked responses. Similar cut-off frequencies have been traditionally used to remove slow trends and higher-frequency noise (and fast modulations of small magnitude) to improve the visualization of EEG evoked responses in previous research (Jacques and Rossion 2007; Nozaradan et al. 2018; Quek et al. 2018). For broadband responses, the envelope of the preprocessed EMG signals (i.e., filtered between 10 and 195 Hz and rectified) was extracted using a Hilbert transform and then lowpass filtered using a fourth-order Butterworth filter with a 5-Hz cut-off frequency to maximize the sensitivity to slow modulations. The envelope was used to capture global changes in broadband EMG signals (Bourguignon et al. 2017; Colon et al. 2017) and determine whether there was any modulation in participants’ muscular activity induced by the stimulus presentation despite being instructed to maintain a constant finger pressure.

For the analysis of both EEG (0.3–30-Hz filtered signals) and EMG (5-Hz lowpass-filtered envelope of the rectified 10–195-Hz signals) broadband responses, epochs of 1 s for 1-Hz trials and 0.5 s for 2-Hz trials, starting 0.5 s before stimulus onset for 1-Hz trials and 0.25 s before stimulus onset for 2-Hz trials, were extracted (see Fig. 1). The 2 first epochs of the 16 stimulus epochs of each trial were removed to avoid transient responses related to the onset of the stimulus sequence. The remaining epochs of all trials in each condition (168 epochs in total, 14 epochs × 12 trials) were then averaged to obtain within-cycle EEG and EMG broadband responses in the time domain and examine cerebral and muscular amplitude modulations induced by stimulus presentation.

#### Beta Band Responses

For beta band responses, a time-frequency analysis was conducted on preprocessed EEG (0.1-Hz high-pass filtered) and EMG (10–195-Hz bandpass filtered and rectified) data in Fieldtrip to compute the power of EEG and EMG signals and the synchronization between the 2 (EEG–EMG coherence). A 250-ms fixed-length sliding window with 10-ms steps from the beginning to the end of each trial was used to compute the power between 0 and 48 Hz for all EEG electrodes and the EMG, and the cross-spectra between each EEG electrode and the EMG, which was needed to compute the coherence. The fixed-length window size of 250 ms, resulting in a frequency resolution of 4 Hz (yielding 12 frequency bins for the 0–48-Hz range), was chosen to avoid overlap between 2 consecutive stimuli and to make it possible to examine within-cycle beta power and coherence modulations with sufficient temporal resolution in both 1- and 2-Hz trials. A multitaper approach, as implemented in Fieldtrip and previous studies that investigated cortico-muscular coherence, was used to compute the power and cross-spectra over time (Mitra and Pesaran 1999; Hari et al. 2014; Piitulainen et al. 2015; Bourguignon et al. 2017). Three tapers in total were used, resulting in a spectral smoothing of ±6 Hz. The power and cross-spectra were computed over time from the beginning to the end of each trial, including the 8 control cycles (no stimuli) before and after the 16 stimulus cycles, for the 12 trials in each condition (see Fig. 1). Stimulus cycles, as for the epochs, corresponded to 1 s for 1-Hz trials and 0.5 s for 2-Hz trials, starting 0.5 s before stimulus onset for 1-Hz trials and 0.25 s before stimulus onset for 2-Hz trials. The power and cross-spectra computed for the 2 first cycles of the 16 stimulus cycles were removed to avoid transient responses related to the onset of the stimulus sequence. The first cycle of the first and last 8 control cycles were also removed to avoid transient responses due to the start of the trial and the stop of the stimuli, respectively, and to have the same number in total of stimulus and control cycles.

This procedure resulted in a total of 168 stimulus and 168 control time-frequency epochs (14 epochs × 12 trials) for each participant, and each modality and tempo condition. For each epoch (1 s long for 1-Hz trials and 0.5 s-long for 2-Hz trials), there was a time-frequency map with the autospectral density for each EEG channel and the EMG channel, and a time-frequency map with the cross-spectral density for each EEG channel (computed with the EMG channel), both with 12 frequency bins and 100 and 50 time steps for 1- and 2-Hz conditions, respectively. For each participant, and each modality and tempo condition, the 168 time-frequency epochs were averaged together to obtain for each EEG channel and the EMG channel an average time-frequency power map for control cycles (i.e., no stimulus presented) and for stimulus cycles. For each participant, and each modality and tempo condition, the 168 time-frequency epochs were used to obtain for each EEG channel the EEG–EMG coherence at each time step computed at a frequency *f* as:

(1) \begin{equation*} {\mathrm{Coh}}_{\mathrm{EEG}-\mathrm{EMG}}(f)=\frac{\left|{S}_{\mathrm{EEG}-\mathrm{EMG}}(f)\right|}{\sqrt{S_{\mathrm{EEG}}(f){S}_{\mathrm{EMG}}(f)}} \end{equation*}

where *S*_EEG_ (*f*) and *S*_EMG_ (*f*) correspond to the autospectral density of the EEG and EMG channels, and *S*_EEG–EMG_ (*f*) corresponds to the cross-spectral density (Bastos and Schoffelen 2016). This procedure resulted in one time-frequency coherence map with 12 frequency bins and 100 and 50 time steps for 1- and 2-Hz conditions for each participant and condition (see grand-average maps in Fig. 6).

To make sure that changes in coherence originated from actual changes in EEG–EMG synchronization and not artificially from time-locked power modulations with systematic changes in the phase of EEG and/or EMG induced by stimulus presentation, EEG–EMG coherence was also calculated on surrogate data (Hari et al. 2014; Bourguignon et al. 2017). For each participant and each condition, EMG trials were permuted in such a way that EEG data were randomly paired with EMG data from another trial. 100 permutations were done in total, resulting in 100 time-frequency coherence maps that were then averaged to obtain one map for each participant and condition.

### Statistical Analyses

For statistical analyses and data visualization, power and coherence in the beta range were obtained by averaging values from 12 to 40 Hz. This range was selected to capture the range of frequencies at which EEG–EMG coherence occurred across all participants (see Fig. 3 showing variability in the frequency range in which coherence was observed across participants). Statistical analyses on EEG–EMG beta coherence were conducted using the average of the electrodes C1 and C3, consistent with the topography of maximal EEG–EMG beta coherence values (see Fig. 3) and compatible with activity from motor cortical sources, as in previous studies (Chakarov et al. 2009; Hari et al. 2014; Mehrkanoon et al. 2014; Bourguignon et al. 2017). The average of the C1 and C3 electrodes was therefore also used to examine broadband and beta band amplitude modulations, assuming amplitude modulations observed on these 2 electrodes would reflect activity originating from these same cortical regions. Amplitude in broadband and beta band was also examined using the average of the FCz and Fz electrodes and the O1 and O2 electrodes, in line with the topographies of broadband and beta power responses presented in Figures 4 and 5, which are assumed to reflect cortical responses to auditory and visual stimuli, respectively (Jacques and Rossion 2007; Nozaradan et al. 2018; Quek et al. 2018).

One set of statistical analyses on broadband and beta band data examined time-averaged responses, and another set examined dynamic responses. In analyses of time-averaged responses, broadband and beta band responses were averaged along the time dimension to test general differences in the amplitude of EEG, EMG, and EEG–EMG coherence across the different modality and tempo conditions, including both control cycles (no stimulus presented) and stimulus cycles. The analyses of broadband and beta band dynamic responses addressed fluctuations in the amplitude of EEG, EMG, and EEG–EMG coherence over time within stimulus cycles (i.e., during stimulus presentation) for the different modality and tempo conditions.

#### Time-Averaged Responses

For time-averaged responses, broadband and beta band responses were averaged across the different time steps of the stimulus cycles and the control cycles to examine global amplitude differences in the different modality and tempo conditions. For broadband responses, time-averaged EMG data (i.e., averaged across the 1000 samples for 1-Hz epochs and 500 samples for 2-Hz epochs) were submitted to a repeated-measures ANOVA with the factors modality (audio, visual, and audio–visual), tempo (1 and 2 Hz) and stimulation (control and stimulus). No statistical analyses were conducted on evoked responses during stimulus cycles in the broadband EEG data, shown in Figure 4 and extensively demonstrated in previous research (e.g., Jacques and Rossion 2007; Nozaradan et al. 2018). For beta band responses, EMG beta power, EEG beta power, and EEG–EMG coherence were averaged across the 100 time steps for 1-Hz trials and the 50 time steps for 2-Hz trials of the time-frequency maps to obtain time-averaged responses. For EMG beta power, time-averaged responses were entered into a repeated-measures ANOVA with the factors modality (audio, visual and audio–visual), tempo (1 and 2 Hz), and stimulation (control and stimulus). For EEG beta power, the factor region (motor [C1 C3], visual [O1 O2], and auditory [FCz Fz]) was added to the factors modality, tempo, and stimulation to test for differences between the different cortical regions. For EEG–EMG coherence, time-averaged responses for motor areas (average of C1 and C3) were submitted to a repeated-measures ANOVA with the factors modality (audio, visual, and audio–visual), tempo (1 and 2 Hz), stimulation (control and stimulus), and permutation (real and permuted). The permutation factor allowed us to evaluate whether differences were genuinely attributable to changes in cortico-muscular coupling rather than to stimulus conditions inducing changes in EEG and/or EMG phase, which might artificially lead to higher coherence values.

#### Dynamic Responses

For dynamic responses, broadband and beta band data were analyzed over the course of stimulus cycles to test for the occurrence of dynamically amplitude-modulated broadband, beta power and beta coherence responses and differences across the different modality and tempo conditions. One- and two-Hertz conditions were analyzed separately, as they have different time steps and potentially different dynamics. The ANOVAs were conducted on demeaned data and focused only on the effect of Time or interactions including this factor. Repeated-measures ANOVAs with the factors modality (audio, visual, and audio–visual) and time (one hundred 10-ms steps for 1 Hz and fifty 10-ms steps for 2 Hz) were conducted on EMG broadband and beta power, and EEG–EMG motor coherence for the 1- and 2-Hz conditions. EMG broadband data were downsampled to 100 Hz when submitted to this ANOVA. The factor region (motor [C1 C3], visual [O1 O2], and auditory [FCz Fz]) was added in the ANOVA on EEG beta power to test for differences between cortical regions that were expected to occur with the presentation of auditory and visual stimuli.

To test the occurrence of significant modulations in dynamic responses over time further, we used cluster-based permutation analyses (Oostenveld et al. 2011). We ran point-by-point one-sample *t*-tests on demeaned data for each of the 6 conditions to test for significant (negative and positive) deviations from 0. Then, we determined clusters of adjacent time points above the critical *t*-value for a parametric two-sided test and the magnitude of each cluster by calculating the sum of the absolute *t*-values constituting each cluster. We used 1000 random permutations (random sign changes) of each participant’s dynamic responses to obtain a reference distribution of maximum cluster magnitude. The proportion of random partitions that resulted in a larger cluster-level statistic than the observed one (*P* value) was calculated. Clusters in observed data were considered as significant if their magnitude exceeded the threshold of the 95th percentile of the permutation distribution. Cluster-based permutation analyses entailing point-by-point one-way ANOVAs with the factor Modality were also used where necessary to compare the magnitude of the deviations between the 3 conditions separately for each tempo.

ANOVAs and cluster-based permutation analyses were also conducted on “permuted” coherence data when significant effects were found on “real” coherence data to confirm that the effects were genuinely attributable to changes in cortico-muscular coupling rather than EEG and/or EMG phase changes induced by stimulus presentation. These analyses were also conducted on permuted data obtained from a single permutation rather than the average of 100 permutations, as the former might decrease the magnitude of random deviations that could occur. No statistical analyses were conducted on evoked responses in broadband EEG data, as these dynamic amplitude modulations have been demonstrated extensively in previous research (see Fig. 4, Nozaradan et al. 2018; Quek et al. 2018).

All statistical analyses were conducted using R version 3.4.3 and graphics were generated with the package ggplot2 (R Core Team 2013; Wickham 2016). Repeated-measures ANOVAs were performed with the package “afex” version 0.19–1 with Greenhouse–Geisser correction applied when the assumption of sphericity was violated (Singmann et al. 2015). Pairwise contrasts were used to examine the significant effects further, with Bonferroni adjustment for multiple comparisons. Data from one of the 17 participants tested was not kept for further analyses because of technical issues during the EEG recording.

## Results

The first part of this section presents the results for time-averaged broadband and beta responses that tested for global amplitude differences in EEG, EMG and EEG–EMG coherence across the different modality and tempo conditions, and stimulus and control cycles. The second part then presents the results for dynamic broadband and beta responses testing for amplitude modulations within stimulus cycles (i.e., during stimulus presentation) in EEG, EMG and EEG–EMG coherence across the different conditions.

### Time-Averaged Responses

#### EMG Broadband and Beta Power

The ANOVAs on time-averaged EMG broadband and EMG beta power responses indicated no significant main effects of Stimulation, (*F*(1, 15) = 0.95, *P* = 0.35, η*_g_*^2^ < 0.0001, and *F*(1, 15) = 0.95, *P* = 0.35, η*_g_*^2^ < 0.0001, respectively), of Modality, (*F*(2, 30) = 1.53, *P* = 0.24, η*_g_*^2^ = 0.0005, and *F*(2, 30) = 1.53, *P* = 0.24, η*_g_*^2^ = 0.0005, respectively), of Tempo, (*F*(1, 15) = 0.01, *P* = 0.93, η*_g_*^2^ < 0.0001, and *F*(1, 15) = 0.01, *P* = 0.93, η*_g_*^2^ < 0.0001, respectively), or any significant 2-way or 3-way interactions between these 3 factors (all *P* values >0.05). These results suggest that global broadband and beta amplitude in participants’ EMG did not change systematically between control cycles and stimulus cycles or across the different modality and tempo conditions.

#### E‌EG Beta Power

**Figure 2:**
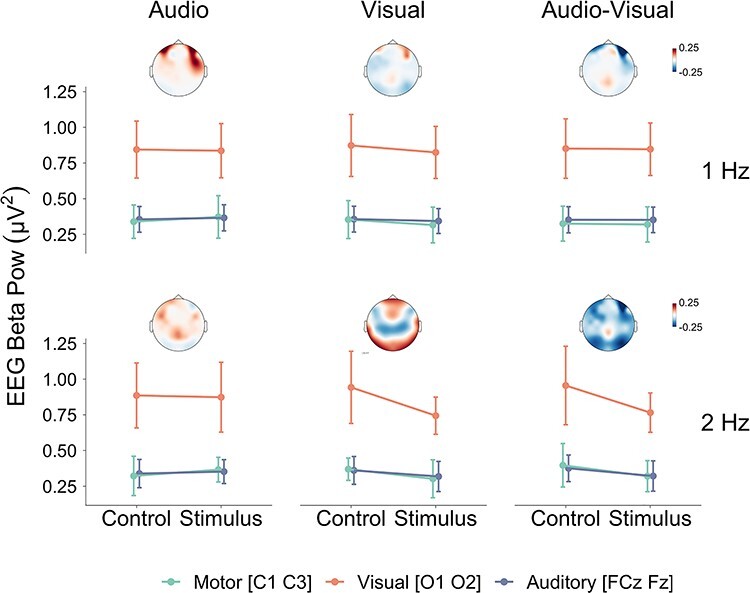
EEG beta (12–40 Hz) power at electrodes compatible with motor, auditory, and visual cortical regions as a function of the different Modality, Stimulation, and Tempo conditions averaged across participants. Error bars represent 1 × 95% CI of the mean computed for within-subject designs (Morey 2008). Topographic plots correspond to Stimulus—Control contrasts averaged across participants.

The ANOVA on time-averaged EEG beta power revealed significant main effects of Region, *F*(2, 30) = 13.04, *P* = 0.002, η*_g_*^2^ = 0.20, Stimulation, *F*(1, 15) = 7.98, *P* = 0.01, η*_g_*^2^ = 0.001, and significant (or close to significant) interactions between Tempo and Stimulation, *F*(1, 15) = 9.90, *P* = 0.0009, η*_g_*^2^ = 0.007, Region and Stimulation, *F*(2, 30) = 4.36, *P* = 0.05, η*_g_*^2^ = 0.0009, Modality and Stimulation, *F*(2, 30) = 4.84, *P* = 0.03, η*_g_*^2^ = 0.001, Tempo, Stimulation and Region, *F*(2, 30) = 4.18, *P* = 0.05, η*_g_*^2^ = 0.0004, and Tempo, Stimulation and Modality, *F*(2, 30) = 3.04, *P* = 0.08, η*_g_*^2^ = 0.0005. As seen in Figure 2, these results indicate lower EEG beta power in occipital areas when visual and audio–visual sequences were presented compared to control, but only at 2 Hz. Pairwise comparisons with Bonferroni correction (18 comparisons in total) yielded significant differences between control and stimulus for the Visual region [O1 O2] in the Visual condition, *t*(183.66) = 5.83, *P* < 0.0001, *d* = 0.27, and audio–visual condition, *t*(183.66) = 5.57, *P* < 0.0001, *d* = 0.25, with 2-Hz sequences. No other pairwise comparisons were significant (all *P* values >0.05). The ANOVA did not reveal any other significant effects (all *P* values >0.05).

#### E‌EG–EMG Beta Coherence

**Figure 3:**
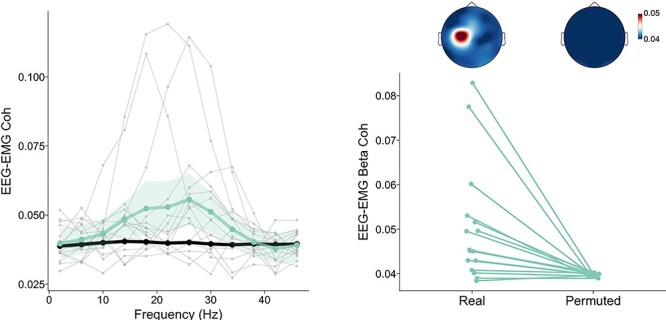
EEG–EMG coherence over cortical motor region [C1 C3] for real and permuted data. The left panel represents grand-averaged real coherence, grand-averaged permuted coherence, and individual real coherence for each participant, for all frequency bins averaged over time and across all conditions. Shaded areas represent 1 × 95% CI of the mean computed for within-subject designs (Morey 2008). The right panel represents the same individual real and permuted data averaged within the beta range (12–40 Hz) with the corresponding grand-averaged topographies.

EEG–EMG coherence over motor [C1 C3] regions exhibited maximal magnitude around 25 Hz, with pronounced variability between participants within the 10–40 Hz range, as depicted in Figure 3. The ANOVA on time-averaged EEG–EMG beta motor [C1 C3] coherence averaged within this frequency range indicated a significant main effect of Permutation, *F*(1, 15) = 10.35, *P* = 0.006, η*_g_*^2^ = 0.21, showing that EEG–EMG coherence over motor areas was larger in real data than permuted data (see Fig. 3 right panel). The ANOVA did not yield other significant main effects or interactions (all *P* values >0.05), which indicates that the global magnitude of EEG–EMG beta coherence recorded over motor areas was not influenced by the stimuli presented and their modality and tempo.

### Dynamic Responses

#### Broadband EEG and EMG

**Figure 4:**
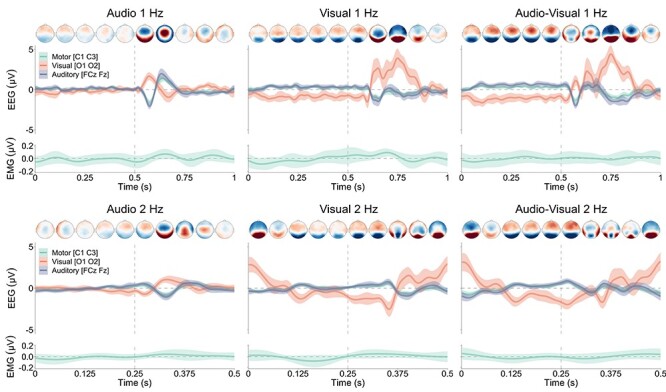
Demeaned EEG (0.3–30 Hz) and EMG (5-Hz lowpass-filtered envelope of the rectified 10–195-Hz signal) for audio, visual, and audio–visual 1- and 2-Hz conditions averaged across participants. Shaded areas represent 1 × 95% CI of the mean computed for within-subject designs (Morey 2008). Note the scaling difference in the time axis for the 1- and 2-Hz conditions, corresponding to 1 and 0.5 s, respectively. Grand-averaged topographies are presented, ranging from −1 μV (blue) to 1 μV (red), for all Modality conditions averaged within 100-ms intervals for 1-Hz conditions and 50-ms intervals for 2-Hz conditions. The vertical dashed line represents the onset of the audio and/or visual stimulus.

As seen in Figure 4, no dynamic within-cycle modulation occurred in broadband EMG in any of the modality and tempo conditions. The ANOVAs on 1- and 2-Hz broadband EMG data indicated no effects of Time (*F*(99, 1485) = 1.17, *P* = 0.13, η*_g_*^2^ = 0.02, for 1 Hz, and *F*(49, 735) = 1.26, *P* = 0.12, η*_g_*^2^ = 0.02, for 2 Hz) or interaction between Modality and Time (*F*(198, 2970) = 0.49, *P* = 0.99, η*_g_*^2^ = 0.02, for 1 Hz, and *F*(98, 1470) = 0.64, *P* = 0.99, η*_g_*^2^ = 0.03, for 2 Hz). Cluster-based permutation analyses did not indicate significant deviations from 0 in any of the 6 conditions, further suggesting the absence of systematic within-cycle modulations in participants’ EMG activity despite the presentation of audio and visual stimuli. Figure 4 shows that classical EEG evoked responses were observed following audio and visual stimulus presentation. No statistical analyses were conducted on these EEG broadband responses, which showed clear dynamic amplitude modulations in line with previous research (Jacques and Rossion 2007; Nozaradan et al. 2018).

#### EMG Beta Power

**Figure 5:**
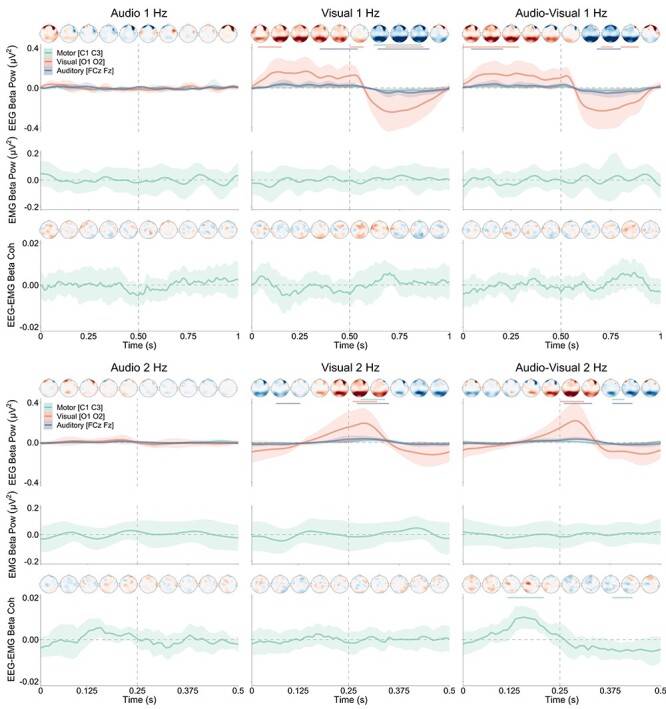
Demeaned EEG power, EMG power, and EEG–EMG motor [C1 C3] coherence in the beta (12–40 Hz) frequency range for the different tempo and modality conditions. Shaded areas represent 1 × 95% CI of the mean computed for within-subject designs (Morey 2008). Note the scaling difference in the time axis for the 1- and 2-Hz conditions, corresponding to 1 and 0.5 s, respectively. Grand-averaged topographies are presented for all Modality conditions between −0.15 (blue) and 0.15 (red) for EEG beta power and −0.015 (blue) and 0.015 (red) for EEG–EMG beta motor coherence averaged within 100-ms intervals for 1-Hz conditions and 50-ms intervals for 2-Hz conditions. The horizontal color lines represent the significant clusters and the vertical dashed line represents the onset of the audio and/or visual stimulus.

The ANOVAs on EMG beta power in 1- and 2-Hz conditions did not reveal any significant main effect of Time (*F*(99, 1485) = 0.72, *P* = 0.98, η*_g_*^2^ = 0.02, for 1 Hz, and *F*(49, 735) = 4.18, *P* = 0.05, η*_g_*^2^ = 0.0004, for 2 Hz), or interaction between Modality and Time (*F*(198, 2970) = 0.80, *P* = 0.98, η*_g_*^2^ = 0.03, for 1 Hz, and *F*(98, 1470) = 0.69, *P* = 0.99, η*_g_*^2^ = 0.03, for 2 Hz). Cluster-based permutation analyses did not indicate significant deviations from 0 in any of the 6 conditions. These results indicate that beta power in participants’ EMG did not exhibit any systematic within-cycle dynamic modulations in all modality and tempo conditions, as seen in Figure 5.

#### E‌EG Beta Power

The analyses on EEG beta power in 1- and 2-Hz conditions revealed dynamic within-cycle modulations of EEG beta power in the visual and audio–visual conditions. The ANOVAs revealed significant time × region × modality interactions for both 1 Hz, *F*(396, 5940) = 4.72, *P* < 0.0001, η*_g_*^2^ = 0.06, and 2 Hz, *F*(196, 2940) = 4.07, *P* < 0.0001, η*_g_*^2^ = 0.05. ANOVAs conducted on each of the modality condition to examine these effects indicated a significant main effect of time and a time × region interaction for the visual (*F*(99, 1485) = 6.36, *P* < 0.0001, η*_g_*^2^ = 0.18, and *F*(198, 2970) = 5.80, *P* < 0.0001, η*_g_*^2^ = 0.15, respectively) and audio–visual (*F*(99, 1485) = 6.27, *P* < 0.0001, η*_g_*^2^ = 0.18, and *F*(198, 2970) = 5.34, *P* < 0.0001, η*_g_*^2^ = 0.15, respectively) conditions for 1-Hz sequences. Corresponding effects were found for 2-Hz sequences—Visual (*F*(49, 735) = 4.90, *P* < 0.0001, η*_g_*^2^ = 0.14, and *F*(98, 1470) = 4.47, *P* < 0.0001, η*_g_*^2^ = 0.13, respectively); audio–visual (*F*(49, 735) = 5.20, *P* < 0.0001, η*_g_*^2^ = 0.14, and *F*(98, 1470) = 4.87, *P* < 0.0001, η*_g_*^2^ = 0.14, respectively).

Cluster-based permutation analyses also indicated significant deviations from 0 in these 4 conditions (see Fig. 5). These results show the occurrence of dynamic modulations in EEG beta power induced by the presentation of visual stimuli that originated from occipital regions. The ANOVAs on EEG beta power in 1 and 2-Hz audio conditions indicated no significant main effect of Time or Time × Region interaction (all *P* values >0.05), suggesting that the auditory sequences did not produce dynamic time-locked modulations of beta power, as seen in Figure 5. These results were confirmed by cluster-based permutation analyses, which did not reveal any significant deviation from 0 in these 2 conditions.

#### E‌EG–EMG Beta Coherence

**Figure 6:**
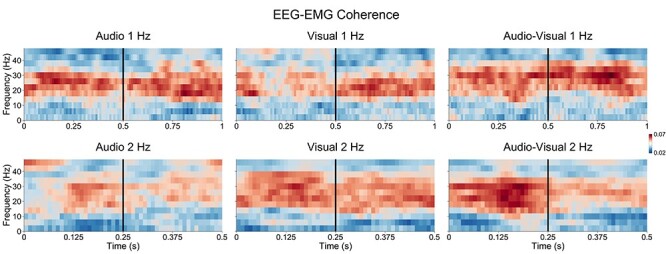
EEG–EMG motor [C1 C3] coherence for all frequencies as a function of the different tempo and modality conditions averaged across all participants. Note the scaling difference in the time axis for the 1- and 2-Hz conditions, corresponding to 1 and 0.5 s, respectively. The vertical black line represents the onset of the audio and/or visual stimulus.

Dynamic EEG–EMG coherence responses recorded over motor areas [C1 C3] for the different tempo and modality conditions are presented in Figure 5 for averaged values within the beta range (12–40 Hz), and in Figure 6 for all frequencies. The ANOVAs on EEG–EMG beta motor coherence in 1- and 2-Hz conditions indicated dynamic within-cycle modulations for the 2-Hz tempo but not the 1-Hz tempo. The ANOVA on 1-Hz motor coherence data indicated no significant main effect of Time, *F*(99, 1485) = 0.92, *P* = 0.69, η*_g_*^2^ = 0.02, or interaction between Time and Modality, *F*(198, 2970) = 0.49, *P* = 0.99, η*_g_*^2^ = 0.02. The ANOVA on 2-Hz motor coherence data yielded a significant main effect of time, *F*(49, 735) = 3.43, *P* < 0.0001, η*_g_*^2^ = 0.07, and a near-significant interaction between time and modality, *F*(98, 1470) = 1.23, *P* = 0.07, η*_g_*^2^ = 0.05.

ANOVAs on each of the 3 modality conditions for 2-Hz data revealed a significant main effect of time for audio–visual, *F*(49, 735) = 4.73, *P* < 0.0001, η*_g_*^2^ = 0.24, but not for audio, *F*(49, 735) = 0.91, *P* = 0.66, η*_g_*^2^ = 0.06, or visual, *F*(49, 735) = 0.22, *P* = 0.99, η*_g_*^2^ = 0.01. These results were confirmed by cluster-based permutation analyses conducted on each of the 6 conditions that indicated significant clusters only for the audio–visual 2-Hz condition (see Fig. 5). The cluster-based permutation analyses and ANOVAs on permuted 2-Hz data in the audio–visual condition indicated no significant clusters and main effect of time, *F*(49, 735) = 0.41, *P* = 0.99, η*_g_*^2^ = 0.03 for permuted data with 100 permutation and *F*(49, 735) = 0.61, *P* = 0.98, η*_g_*^2^ = 0.01 for permuted data with 1 permutation. This confirms that the dynamic modulation of EEG–EMG motor coherence in this condition is attributable to a genuine increase in cortico-muscular coupling rather than systematic changes in the phase of EEG and/or EMG induced by stimulus presentation artificially inflating coherence values. Furthermore, it can be noted that the increase of EEG–EMG coherence was maximal about 100 ms before the presentation of the stimulus, in contrast to the increase of EEG beta power that was maximal after the stimulus, and that this increase in EEG–EMG coherence was maximal over motor areas as shown on the grand-averaged topographies in Figure 5. A cluster-based permutation analysis that compared the amplitude of the modulations in the 3 conditions at 2 Hz did not indicate significant clusters. Direct evidence of larger modulation in the bimodal condition than the unimodal conditions is therefore lacking, even though only the bimodal condition displayed significant modulation over time.

## Discussion

This study investigated concurrent EEG and EMG responses of participants passively listening to sequences of auditory tones and/or observing sequences of visual flashes during isometric muscular contraction. The results revealed that the presentation of the sequences not only resulted in evoked brain responses to the visual and auditory inputs, but also in dynamic modulations of beta band coherence between EEG and EMG recorded from the finger. This cortico-muscular coupling might underlie motor entrainment to environmental rhythms.

Our EEG data indicated the occurrence of evoked responses a few hundred milliseconds after the presentation of periodic auditory and/or visual stimuli for both 1- and 2-Hz conditions, in line with previous research (Jacques and Rossion 2007; Nozaradan et al. 2018; Quek et al. 2018). These responses were accompanied by amplitude modulations in the beta frequency band for visual and visual–auditory stimuli but not for auditory stimuli. In both conditions, these modulations appeared to be of larger magnitude in occipital regions, suggesting modulations in visual activity. Although EEG beta modulations with audio stimuli have recently been reported (Chang et al. 2018, 2019), there is evidence that the cortical tracking of audio signals is more readily observed with MEG than EEG under some circumstances (Destoky et al. 2019), which may partially explain our result. We also did not find dynamic changes in participant’s index finger related EMG activity across all frequencies and in the beta frequency band in particular. Nevertheless, the results revealed that the synchronization of beta band oscillations between muscular activity and EEG activity recorded at electrodes over cortical motor regions was dynamically modulated when audio–visual rhythms were presented. This EEG–EMG coherence increased prior to stimulus onsets, in contrast to EEG evoked sensory responses occurring after stimulus onsets.

This finding extends previous research by showing that dynamic modulations in cortico-muscular coherence do not only occur following an unexpected stimulus (Piitulainen et al. 2015) but also persist with periodic stimuli, highlighting the potential role of these modulations in neurophysiological processes underlying motor entrainment to environmental rhythms. The observed dynamic modulations of EEG–EMG beta coherence were tempo and modality specific. The modulations were found for 2-Hz sequences, but not for 1-Hz sequences, when both auditory and visual stimuli were presented together. This result is in line with the hypothesis that audio–visual 2-Hz rhythms would result in the strongest entrainment. It has been previously reported that 2 Hz is the preferred tempo for rhythm production and perception, and it has been speculated that this may be due to biomechanical properties of human locomotion (MacDougall and Moore 2005; Large 2008; Todd and Lee 2015; Bouvet, Varlet, Dalla Bella, Keller, Bardy 2019, Bouvet, Varlet, Dalla Bella, Keller, Zelic, et al. 2019). It can be noted also that only 2-Hz stimuli resulted in overall beta power decreases in EEG compared to when no stimuli were presented in control cycles, which might also reflect the stronger influence of this particular tempo on brain activity. Benefits of audio–visual stimuli over unimodal stimuli are also in accordance with results previously reported in multisensory integration literature, including in the context of movement synchronization where timing processing improves with the availability of cues from more than one modality (Stein and Meredith 1993; Elliott et al. 2010, 2011; Eaves et al. 2019). It will remain nevertheless necessary to confirm this bimodal advantage in future studies, as cluster-based permutation testing that directly compared the amplitude of the dynamic modulations in the 3 conditions at 2 Hz did not indicate significant differences, even though only the bimodal condition showed a significant modulation over time.

Furthermore, previous research has shown that human movements can entrain to rhythms that are unimodal and have tempi slower than 2 Hz (Richardson et al. 2007; Lopresti-Goodman et al. 2008; Burger et al. 2018; Varlet, Williams, and Keller 2020), suggesting that dynamic modulations of EEG–EMG beta coherence might be expected to occur in these conditions under some circumstances. The sequences of simple pure sine tones and/or visual flashes used in the current study might not have favored the occurrence of motor entrainment under unimodal conditions and at the relatively slow 1-Hz tempo. Musical properties of auditory rhythms such as the saliency of the beat or the degree of syncopation, as well as simple properties such as the pitch of the sounds, are known to modulate the strength of motor entrainment (Burger et al. 2013, 2018; Stupacher et al. 2013; Etani et al. 2018; Lenc et al. 2018). Enhanced movement entrainment and EEG tracking of auditory rhythms have been found with low-pitch sounds (Hove et al. 2014; Lenc et al. 2018; Varlet et al. 2020), suggesting that using lower-pitched sounds could have resulted in larger effects on EEG–EMG beta coherence. Visual rhythms are also not all equal in producing spontaneous movement entrainment. Properties such as their continuity, amplitude, and movement velocity profile modulate the occurrence and strength of visuomotor entrainment (Varlet, Coey, et al. 2012; Varlet et al. 2014; Zelic et al. 2016, 2018). The biological naturalness of visual rhythms can also play a key role, with both human-like kinematics and appearance facilitating entrainment (Kilner et al. 2003; Press et al. 2011). Therefore, the specific properties used in the current study (i.e., discrete 500-Hz pure sine tones and flashing dots) might not have been conducive to motor entrainment, especially in unimodal and 1-Hz conditions. To address this, future studies could test the effects of other types of visual and auditory rhythms to further understand the range of stimuli and tempi that produce spontaneous changes in beta band cortico-muscular coupling.

The dynamic modulation of EEG–EMG beta coherence was characterized by an increase in coherence about 100 ms before the occurrence of each audio–visual stimulus. This temporal profile seems to suggest an anticipatory motor response that, if participants were free to move, could support the production of overt movement synchronized with the stimulus. The observed profile might also reflect the involvement of motor related areas in rhythm perception, where anticipatory amplitude modulations of beta band neural oscillations have been observed using MEG (Press et al. 2011; Fujioka et al. 2012, 2015; Large et al. 2015; Morillon and Baillet 2017). Motor related areas are involved in supporting the prediction of upcoming events during rhythm perception and the increase of coherence in beta band neural oscillations between EEG and EMG activities might capture these mechanisms. However, an alternative explanation could be that the increase of EEG–EMG coherence before the stimulus might actually be a response to the previous stimulus in the sequence. Even if such a response is not an anticipatory response, it could still support the production of overt movement synchronized with the stimulus. To disentangle these 2 hypotheses and confirm the anticipatory nature of such EEG–EMG coherence increases, it would be important in future studies to manipulate the interstimulus intervals (i.e., the tempo) around 0.5 s to examine whether the anticipation remains constant independently of these manipulations (Fujioka et al. 2012; Merchant et al. 2015). The link between the EEG–EMG coherence response and actual movement production might also be investigated in future studies by comparing the dynamics of EEG–EMG beta coherence modulations and the movements actually produced by participants when they are free to move, such as tapping a finger at their preferred tempo (Repp 2005). The strength of the entrainment in EEG–EMG coherence and actual movements could be compared across experimental conditions and participants to confirm the link between the 2, with larger EEG–EMG coherence modulations correlating with stronger movement entrainment taken as evidence for a functional association.

More generally, as EEG–EMG coherence might capture the neurophysiological processes underlying motor entrainment, this measure could be of particular interest in a range of applied research fields (Kelso 1995; Phillips-Silver et al. 2010; Kugler and Turvey 2015). EEG–EMG coherence could help to better understand how humans respond to continuously changing environmental constraints in real-world contexts. Producing or dancing with music, for instance, requires the adaption to and anticipation of complex auditory and visual rhythms (Phillips-Silver and Keller 2012; MacRitchie et al. 2017). Furthermore, one needs to continuously adapt movements across different time scales and body segments. EEG–EMG coherence could help in future research to better understand the neurophysiological processes supporting the production of such complex rhythmic patterns (Mima and Hallett 1999; Grosse et al. 2002; Boonstra 2013). EEG–EMG coherence might also be fruitful in probing the processes underlying abnormal movement entrainment. Indeed, abnormal movement entrainment to environmental rhythms has been reported with a wide range of pathologies, from neurological to social disorders (Varlet, Marin, Raffard, et al. 2012; Del-Monte et al. 2013; Hove and Keller 2015). EEG–EMG coherence could provide neuromarkers that could ultimately help to offer individualized interventions based on objective neurophysiological criteria.

It is important to note that EEG–EMG coherence measures can also have limitations (Bastos and Schoffelen 2016). In the current study, the main limitation is that changes in EEG–EMG coherence were only captured if they were time-locked across stimulus cycles and trials. This is often the case with coherence, as it requires a large number of trials to be accurately calculated. It is nevertheless possible that other less systematic changes in cortico-muscular connectivity might have occurred and been influenced by the different sequences presented. Future studies should therefore explore other measures of connectivity to further understanding of the dynamic changes in cortico-muscular coupling induced by environmental rhythms.

To conclude, the current study demonstrates that the coherence between activity in cortical motor areas and muscular activity in the beta frequency band is dynamically modulated during the passive listening and observation of environmental rhythms. Despite a lack of changes in EMG amplitude and the occurrence of EEG evoked responses after stimulus onsets, EEG–EMG coherence increases prior to stimulus onsets. The observed dynamic modulations in beta band cortico-muscular coupling induced by periodic and predictable stimuli provide a potential mechanism for motor entrainment. Dynamic EEG–EMG coherence modulations occurred particularly when visual and audio sequences were presented together at 2 Hz, confirming at a neurophysiological level the enhancement of motor entrainment to rhythms that are multimodal and close in tempo to the preferred frequency of human movement. These findings demonstrate the potential of EEG–EMG coherence as a measure for furthering the understanding of the neural mechanisms in motor related brain areas supporting rhythm perception and production in humans.

## Notes

The authors thank Donovan Govan for his technical assistance and Alanna Wade for her assistance with data collection. *Conflict of Interest:* None declared.

## Funding

Australian Research Council (DP170104322, FT140101162, DE160101064).
